# Supplementary material for: How can instructions and feedback with external focus be shaped to enhance motor learning in children? A systematic review
Source: PLoS One. 2022 Aug 25;17(8):e0264873. doi: 10.1371/journal.pone.0264873 (PMC9409566; doi:10.1371/journal.pone.0264873)
Supplement: Attachment — Submitted filename: Peer review PLOS - systematic review EF children.docx [file pone.0264873.s004.docx]

This paper presents the results of a systematic review on the effects of instructions and feedback with an external focus of attention on motor learning in children. It focusses on the manipulation of frequency, timing and form and its effects on the performance of functional skills. Based on the best-evidence synthesis, the authors cautiously conclude that there is some evidence for using self-controlled feedback, visual instructions and a continuous frequency of feedback. Improving motor skill learning in both typically and a-typically developing children is both a scientifically and practically relevant topic. Therefore, the review can potentially contribute to the field. However, I do have some questions and suggestions, mostly related to the theoretical frameworks and interpretations of the findings and with that also question the conclusions that are drawn.

**Major issues/comments**

Introduction

1. The authors introduce the external focus (EF) of attention in relation to implicit learning. While there are indeed indications that an EF indeed leads to a more implicit learning process, the majority of the studies on EF (at least when compared to an internal focus) use a different theoretical framework (constrained action hypothesis). I would suggest that the authors include a more elaborate theoretical framework, acknowledging the different ‘lines of research’ on this topic which can strengthen their argumentation for the specific research questions and hypotheses.
2. In addition to this point I would suggest that the authors briefly explain why they only focus on an EF and on aspects that can be controlled by an instructor (frequency, timing and form). For example, the authors also mention that an IF might be more beneficial is specific populations (line 65), and mention the moderating effect of child and task characteristics (line 78).
3. A final comment regarding the introduction would be to strengthen the argumentation for the hypotheses that are given at the start of the method section. In the paragraph starting at line 68 a very brief explanation of the specific ‘instructor controlled’ aspect of EF feedback, but I would prefer to know more about the how and why. For example, in line 73-74 the authors mention that ‘*Self-controlled feedback may enhance children’s motor learning more than instructor-controlled feedback’*. Why is this the case, what is the mechanism? And is this specifically related to an EF

Methods

4. Given the different theoretical backgrounds that studies on EF may have, I would suggest to include a clear definition as to what the authors include as an EF. For example, in the included studies I saw many that use knowledge of results as a form of feedback, which may not be regarded as an EF when compared to the literature that use the constrained action hypothesis as a theoretical framework.
5. I agree with the authors that a meta-analysis on the data is not possible and their decision to perform a best-evidence synthesis. I do, however, want to question the decision to combine the results of typically developing and a-typically developing children in their rating of the evidence for a parameter of interest. The authors may want to revisit this decision, or at least provide a rationale and discuss the implications in the discussion, as this greatly influences the interpretation of the findings and the conclusions.

Results

6. Did you, or the literature that you are reviewing, consider to also include if groups improve their performance over time instead of only focusing on group differences at a specific time point? I would argue that if children do not improve their performance (which may be the case with some very short practice periods) it is not surprising that group differences are also lacking. Therefore, this lack of a significant effect may not be a good representation of the effect that you are most interested in (specific feedback parameters)

Discussion

7. As also mentioned in comment 5, I question the interpretation of the findings when they are based on a combination studies on both typically- and a-typically developing children. For example, the majority of the studies (5 total, ) found a beneficial effect on retention for reduced frequency, and only one study showed better retention with continuous frequency, but in children with ASD, leading to conflicting evidence. On the other hand, limited evidence for continuous frequency, as opposed to reduced frequency, is claimed with only one study performed for movement quality. Also, the suggestion that visual feedback might be preferred is based on 1 study in children with MID and ASD. I believe the discussion should be better balanced by acknowledging how characteristics of theses specific groups may have influenced the outcomes of the individual studies and with that the overall interpretation of the evidence.
8. Overall, the discussion had a large focus on the practical value of the outcomes, but is missing the more theoretical explanations an implications for the (inconsistent) results, which links to my comments about the theoretical framework in the introduction (comment 1 and 3).
9. In lines 343-350 the authors describe the outcomes of a previous systematic review on the frequency of feedback on motor learning. Given the arguments presented here, I would question if the hypothesis that the authors have stated regarding the benefits of reduced feedback is correct?
10. The suggestion made in lines 362-365 should be placed a bit more cautiously. That is, while there are some benefits shown for self-controlled feedback, 2 studies compare this with a yoked condition. It can be argued that the moments when an instructor provides feedback, are more helpful than the moments a experimental counterpart chose this. Also, according to table 1 the study of Hemayattalab (2014) did not show a difference between self-controlled and instructor-controlled feedback, which contradicts your suggestion.
11. In line 403-404 the authors mention that instructors should adapt instructions to the individual (among other things). I agree with this suggestions and given the different populations included in this review (and their specific characteristics) I wondered if the authors could give some informed suggestions about which aspects of the individual instructors may want to focus on

**Minor issues/comments**

Methods

1. Line 122. Tactile instructions are mentioned as an exclusion criteria for the control condition given the IF, but are not all instructions that may promote an IF excluded?
2. I would like some numbers or percentages on the (dis)agreement between authors regarding study selection and methodological quality assessment

Results

3. In table 1 I would suggest to include more information about the specific feedback or instructions that were given, as this is one of the main characteristics of interest in this review.
4. I would like to suggest some re-ordering in the results section. I would prefer the description of the methodological quality a bit earlier in the results. Also, you may want to consider combining the general description of studies focused on a specific parameter (paragraphs starting at lines 230, 239 and 247) with the best-evidence synthesis of that parameter. I was now going back and forth between these sub-sections for a complete overview (and you may also prevent writing info in 2 places).
5. Line 274, the order of the references seems mixed (72, 73, 82-84, 74, 81)?
6. There is an inconsistency between table 1 and 2 regarding the study of Wulf (2010), where table 1 shows no difference between groups, and table 2 does show a difference for movement quality
7. Line 317, the authors mention 33% self-controlled feedback, whereas table 1 shows that the feedback is for 3 out of 10 trials, which should be 30%

Discussion

8. Given the practical value that the authors stress in the discussion, they may want to mention aspects of ecological validity of the studies included in the review in the discussion
